# Measurement of Complex Permittivity for Rapid Detection of Liquid Concentration Using a Reusable Octagon-Shaped Resonator Sensor

**DOI:** 10.3390/mi14030542

**Published:** 2023-02-25

**Authors:** Chun-He Quan, Xiao-Yu Zhang, Jong-Chul Lee

**Affiliations:** 1 JCET Stats ChipPAC Korea, Jayumuyeok-ro, Jung-gu, Incheon 22397, Republic of Korea; 2 Department of Electronic Convergence Engineering, Kwangwoon University, 20 Kwangwoon-ro, Nowon-gu, Seoul 01897, Republic of Korea

**Keywords:** relative complex permittivity, substrate integrated waveguide cavity, liquid concentration, octagon-shaped sensor, radio frequency

## Abstract

Substrate-integrated waveguides (SIWs) are widely used in microwave systems owing to their low cost and ease of integration. In this study, an SIW-based resonator that reacts to the complex permittivity variation of solutions with dimensions of 79.2 mm × 59.8 mm is introduced. This octagon-shaped sensor can be installed on a preliminary monitoring system to test water quality by observing the parameter variations caused by external factors. The resonant structure was used to test different concentrations of ethanol–water and acetone–water mixtures for verification. The resonant frequency and quality factor (Q-factor) were found to vary with the relative complex permittivity of the liquid in the S-band, and the electric field distribution varied when liquid droplets were placed in the center of the substrate. The designed sensor operates at 2.45 GHz in the air, and the observed minimum resonant frequency shift with liquid was 15 MHz. The measurement error was approximately 3.1%, and the results reveal a relationship between the resonant frequency and temperature as well. Considering the observed sources of error, the measured relative permittivity is consistent with the actual values. The proposed sensor is economically convenient and suitable for various test environments.

## 1. Introduction

A sensor is a subsystem, machine, or module used to identify targets or detect changes in environmental variables. With advances in their small size and simple operation, sensors are extensively being applied in electronic and electrical devices. Among the multiple types of sensors available, permittivity sensors are among the most widely used. As a function of state, which depends on the magnitude, frequency, temperature, and many other factors, the permittivity of a material must be accurately measured because it plays a key role in technological fields, including chemistry, physics, and biomedicine. For example, strong buildings can withstand unexpected natural disasters, but an incorrect mixing ratio of compounds may cause a building to collapse. In addition, the robustness of structures can be judged by measuring the relative permittivity of building material samples to ensure quality. In automobiles, long-term non-destructive monitoring of engines helps extend their service life [1,2,3,4]. In microwave applications, permittivity is considered a basic parameter that carries critical information, such as the robustness and electrical energy capacity of a material. 

Several traditional methods have been used to determine the permittivity of materials. The open-ended coaxial probe method is practical for determining permittivity when a target is difficult to reach in its natural environment. The free-space method is realized by simultaneously measuring the phase and amplitude of the transmission wave while changing the length of the material under test (MUT) to calculate its permittivity. Moreover, many other enhanced methods of testing permittivity exist, such as the two-port transmission line method and the phase sensing method [5,6,7,8,9]. Measuring devices are preferred for testing MUTs because they are portable and provide high-precision measurements. Traditional testing waveguides or transmission lines are generally bulky and expensive; therefore, the feasibility of an enhanced chipless radio frequency identification permittivity sensor that works within the industrial scientific medical (ISM) band was suggested [10]. Time-domain reflectometry is applied as the operating method, with several antennas connected to the reader and tag. However, most sensors are specifically designed to measure gases or solids. Moreover, measurement is difficult because of the complicated sensor structure. 

As a result, substrate-integrated waveguide (SIW) technology has been applied in filter and sensor designs in recent years to check water quality. The resonator must be immersed in a large amount of liquid or filled with samples using a micro-capillary to characterize a material accurately [11]. Microfluidic technology has recently emerged as a solution to the wastefulness of specimens [12]. However, this type of sensor is difficult to apply in industrial production. 

An octagon-shaped sensor, which has a simple design and is easy to fabricate, was proposed in this study to address these issues. The eight sides of the octagon constituting the sensor are close to the center slot, enabling variable changes to be observed clearly due to its compact size. An SIW-based structure was introduced to measure the relative complex permittivity of a liquid. The characteristics of the resonant frequency and quality factor (Q-factor) are influenced by the MUT through a rectangular slot on the top metal plane. The operating frequency was set between 2.40–2.49 GHz. Various concentrations of mixtures were prepared, with the sensor tested three times at room temperature for accuracy. This sensor can be installed in various home devices owing to its simple structure and compact size. The rest of this article is organized as follows: Section 2 presents the design of the SIW sensor and the process of planning an experiment to test the relative complex permittivity of liquids. Section 3 describes the measurement results of binary mixtures of ethanol–water and acetone–water. Finally, discussions and conclusions are given in Section 4 and Section 5. 

## 2. Materials and Methods

As key devices in radiofrequency (RF) technology research, waveguides are used to guide waves and reduce losses during propagation. Various structures with easy fabrication and high performance have been used in microwave engineering. An SIW structure prototype was first proposed in the late 20th century. After years of improvement, the SIW is considered a new standard type of waveguide with low radiation loss. The structure is composed of arrayed metallic holes connecting the upper and lower metal layers on a low-loss dielectric substrate that can be connected to circuits and various active and passive microwave devices on the same dielectric substrate. As a result, SIWs are widely used in RF systems and antenna arrays owing to their advantages of low cost and ease of integration. SIWs can be fabricated using printed circuit boards, low-temperature co-fired ceramics, and other manufacturing processes [13].

**Figure 1:**
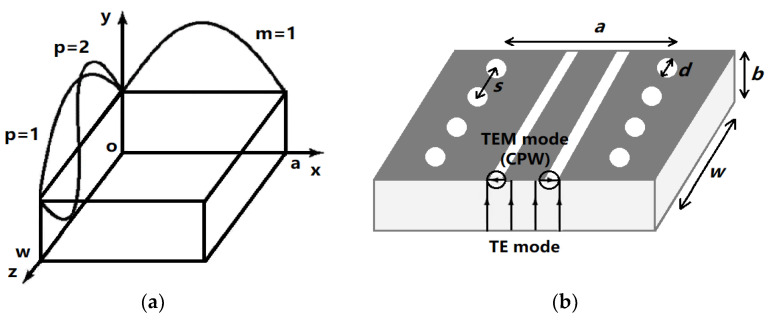
Feeding structure of SIW cavity: (**a**) Electric field distribution of TE_101_ mode and TE_102_ mode; (**b**) Basic structure of SIW cavity with CPW feedline [15].

An SIW resonator with a planar structure was used to measure liquid permittivity. The sidewalls of classical waveguides are composed of metal as an ideal electrical wall, although the gap between vias in an SIW leads to slight radiation loss. However, impedance matching can be improved when the gap between the conductor and ground is sufficiently small for a certain feed length. The transmission characteristics of SIWs are identical to those of classical waveguides. As essential parameters of the waveguide, the lengths of the wide and narrow sides directly affect the working frequency and cut-off frequency of the waveguide. An effective width *a_e_* can be derived by applying waveguide theory to an SIW. The cut-off frequency of the SIW is associated with the width of the waveguide and the distance between the two via rows. The radiation loss of the SIW can be reasonably small if *s*/*d* < 2.5, with *s*/*d* = 2 recommended for the design, where *d* is the diameter of a via and *s* is the distance between two successive vias. Additionally, a relatively high mechanical strength was obtained using the SIW structure with relatively easy fabrication [14]. As shown in Figure 1, a SIW resonator with propagating modes of TE_101_ and TE_102_ was proposed, with a coplanar waveguide (CPW) slot inserted between the vias of the SIW working as the feedline. Coupling effects occurred between the TE mode propagating in the SIW and the TEM mode propagating in the CPW when power was transmitted in the resonator cavity. Various types of cavities with surrounding fences can be designed using the SIW structure, with electric and magnetic field distributions identical to those in a rectangular waveguide. However, only the TE_m0p_ mode can exist in the SIW resonator as the electrical current cannot propagate between vias, unlike in the classical waveguide resonator. The resonant frequency *f* of the TE_m0p_ mode, primarily affected by the length *a* and width *w* of the SIW cavity, can be derived as follows [11]:


(1) $$a_{e}=a-\frac{d^{2}}{0.95s}$$

(2) $$w_{e}=w-\frac{d^{2}}{0.95s}$$

(3) $$f\left(TE_{m0p}\right)=\frac{c}{2\pi \sqrt{\varepsilon_{r}}}\sqrt{{\left(\frac{m\pi }{a_{e}}\right)}^{2}+{\left(\frac{p\pi }{w_{e}}\right)}^{2}}$$

where *a_e_* and *w_e_* denote the equivalent length and width of the SIW cavity, respectively; *m* and *p* denote the mode numbers; *ε_r_* denotes the substrate relative permittivity; and *c* denotes the speed of light in free space. 

The resonator is used in various RF devices, such as filters, oscillators, and amplifiers. The principle of microwave resonators is identical to that of resonators with an equivalent model comprising an RLC parallel resonant circuit. The value of the Q-factor may be affected by the losses in the SIW, including dielectric loss in the substrate, ohmic loss in the metal, the radiation loss caused by the slot, and the internal resistance of external measuring devices such as a network analyzer. The Q-factor, as a dimensionless parameter, describes the underdamping of a resonator or oscillator. The Q-factor is proportional to the sum of the average electric and magnetic energies stored in the resonator and inversely proportional to the cavity loss. The unloaded Q-factor *Q_u_* can be calculated using

(4) $$Q_{u}={\left(\frac{\varepsilon ”}{\varepsilon ’}+\frac{1}{Q_{c}}\right)}^{-1}$$

where *Q_c_* is the Q-factor of the resonator with a lossless dielectric and *ε’* and *ε”* denote the real and imaginary parts of the substrate, respectively [16].

(5) $$Q_{c}=\frac{{\left(kaw\right)}^{3}b\eta_{o}}{2\pi^{2}R_{m}}\frac{1}{\left(2p^{2}a^{3}b+2bw^{3}+p^{2}a^{3}w+aw^{3}\right)}\;$$

(6) $$R_{m}=\sqrt{\pi f\mu_{0}/\sigma }$$

Generally, the thickness of a rectangular waveguide cavity resonator is much larger than that of an SIW resonator. *Q_c_* decreases with decreasing thickness of the resonator. *Q_c_* of the TE_10p_ mode can be determined by (5) when the surface resistance *R_m_* of the metal wall is defined by (6), where *k* denotes the wave number, *σ* denotes the conductivity, and *η_0_ = *$\sqrt{\mu_{0}/\varepsilon_{0}}$ = 377 Ω denotes the impedance of free space [15]. 

The proposed transition structure of the CPW line was introduced between the feed line and the SIW cavity. As the characteristics of the central part of the SIW sensor varied most prominently, a compact octagon-shaped structure was used to keep all sensor parts close to the center and make the feed line and slot lie in a straight line. The resonant frequency and Q-factor of the SIW resonator were primarily determined by the relative permittivity of the substrate. A Taconic TLX-8 series board with a dielectric constant, loss tangent, and thickness of 2.55, 0.0018, and 1.016 mm, respectively, was used for fabrication. The substrate was composed of multilayer glass fibers [17]. 

**Figure 2:**
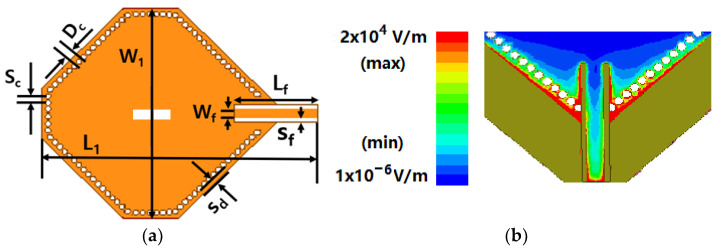
Designed SIW resonator: (**a**) Dimensions; (**b**) Electric field distribution of the proposed SIW cavity.

**Figure 3:**
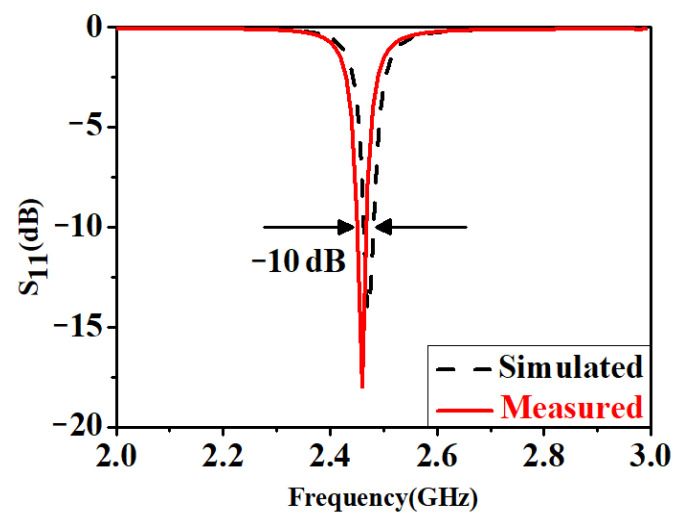
S-parameter of designed resonator.

The dimensions of the designed SIW resonator were optimized by the simulation tool ANSYS High-Frequency Structure Simulator (HFSS) 2021 R1 using radiation boundary condition with the number of mesh more than 100 thousand as shown in Figure 2a, which significantly reduced the cavity volume. The eigenmode solver was also employed to calculate the resonance mode at the S-band to suppress unwanted modes in the waveguide. The dimensions were *S_c_* = 2 mm, *D_c_* = 0.8 mm, *W_1_* = 59.8 mm, *L_1_* = 79.2 mm, *S_d_* = 1.2 mm, *W_f_* = 3 mm, *L_f_* = 25 mm, and *S_f_* = 1 mm. The electric field distribution of the designed feedline at the resonant frequency was calculated using a three-dimensional model drawn from the HFSS, as depicted in Figure 2b. The designed feeding structure could excite the TE_101_ mode without affecting the proximity of the original cavity. Generally, a deviation of 0.1 in the real part of the relative permittivity of the substrate results in a 50–150 MHz resonance frequency shift. An Agilent 8719ES network analyzer, with a step of 10 kHz and a frequency range between 50 MHz–13.5 GHz, was used to measure the SIW resonator. *Q_u_* and the resonant frequencies of the SIW resonators *f_0_* were derived from the measured S-parameter. As depicted in Figure 3, the measured and simulated results show a good agreement with a resonant frequency of approximately 2.45 GHz. The fractional bandwidth of 1.1% with the return loss better than −10 dB in the ISM band was obtained. 

**Figure 4:**
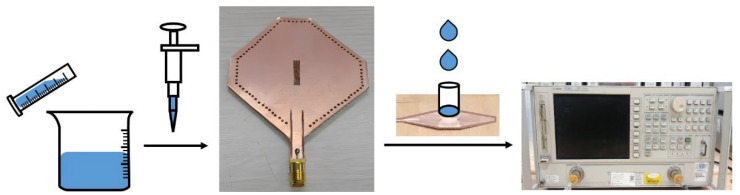
Fabricated SIW sensor under test.

According to the cavity perturbation theory, either a small deformation of the cavity or the introduction of foreign objects will affect cavity performance. Generally, electromagnetic wave attenuation occurs in a planar resonance cavity when a high-loss dielectric material and sensor are in direct contact. The sensor can be partially immersed in a liquid; however, the amount of liquid used for each measurement will vary. Therefore, a quarter-wavelength slot was created at the top plane, where the distribution of the electric field changes rapidly [18]. The performance of this model was stable because only a small area of the sensor with an open structure was in contact with the liquid. As depicted in Figure 4, a narrow slot with a length of 11.8 mm and width of 2.9 mm was fabricated on the sensor. The slot was surrounded by a short plastic hollow tube to prevent liquid spills when conducting the experiments. The solutions were completed with a measuring cylinder and beaker, and a clean pipette tube was used to transfer equal amounts of liquids. The S-parameters tested by the network analyzer vary with the concentration of the mixture because the equivalent complex permittivity of the resonant cavity was affected by the liquid in the slot [19].

A reference temperature of 25 °C was selected and kept constant, considering that the temperature affected the test results. The resonant characteristics of the SIW resonator with deionized water at 30, 25, and 20 °C are listed in Table 1, which shows that *f_o_* and *Q_u_* vary slightly with temperature. An effective method of reducing errors in volume and measurement results involves multiple measurements of the average values. A binary mixed solution of ethanol and water was first selected as the test sample because of its low cost and non-toxicity. The structural and diffusion properties of the mixed solutions have been studied for industrial applications. The mixed solution forms hydrogen bonds, resulting in variations in the physical and chemical properties with different ratios of solutions; therefore, further study on the properties of mixed solutions is necessary. A total of 21 test samples were prepared with a mole fraction of ethanol between 0–100%. The volumes of the mixture constituents are expressed in moles because the volume of the solution may change after mixing due to intermolecular spaces. Water (H_2_O) and ethanol (CH_3_CH_2_OH) were used to prepare the mixture proportioning in 5% intervals after purification and distillation [20]. The capacities of the measuring cylinder, beaker, and pipette tube should not be too large, considering that the liquid used in a single test is generally no more than 1 mL. Every piece of equipment was cleaned after being used to test a sample, and the response time for each sample did not exceed 15 s. 

A model that reflects the relationship between the sensor parameters and the permittivity can be used to obtain the exact value of the complex permittivity of a liquid. An approximate machine-learning model that reflects the relationship between the SIW sensor parameters and relative permittivity was built using Python because it integrates well with open-source libraries. The Python Data Analysis Library was used as an accurate and flexible data manipulation tool to first analyze the data. Next, a decision-tree-based method—extreme gradient boosting—was adopted [21]. This method was selected to deal with distributed problems, microwave theoretical analysis, and calculations owing to its advantages of high-speed dynamic response and memory ability. The learning procedure started by inputting a set of tabulating data consisting of *f_o_* and *Q_u_* extracted from the simulated results, and stopped when the relationship between the calculated relative permittivity and the simulated values of the resonant frequency and Q-factor was mapped [13]. The real and imaginary parts of the relative complex permittivity could be computed by entering the values of the measured *f_o_* and *Q_u_* after establishing a two-input, two-output model. 

## 3. Results

**Figure 5:**
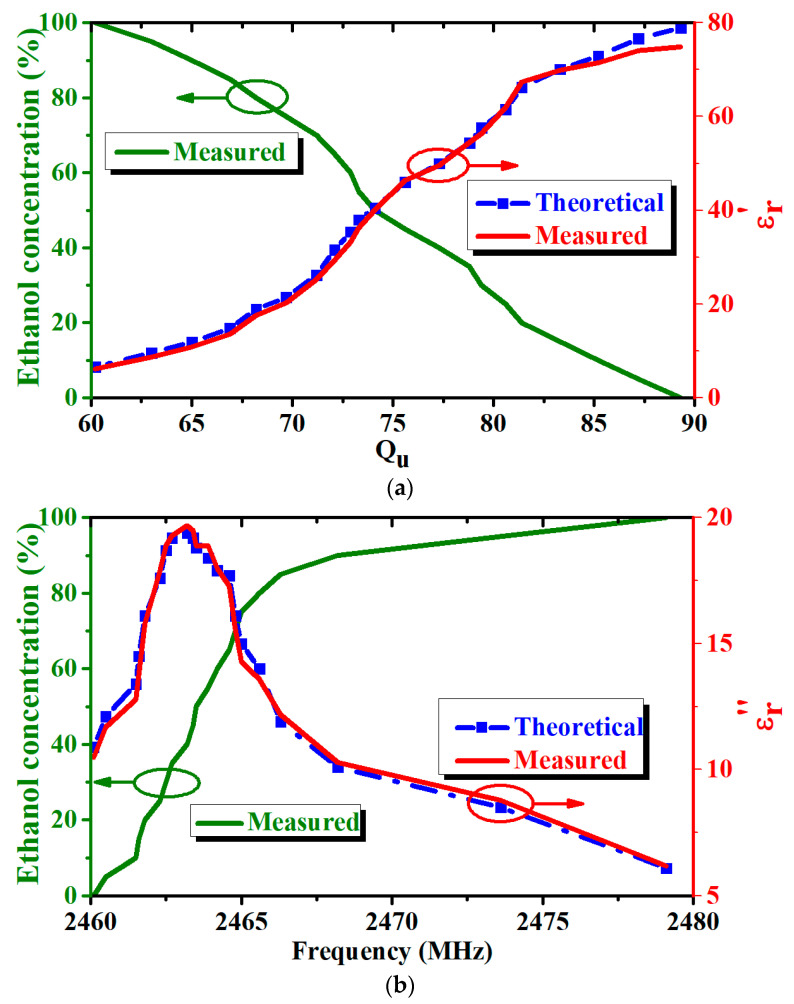
Test results of ethanol–water mixtures: (**a**) Ethanol concentration and *ε’* versus *Q_u_*; (**b**) Ethanol concentration *ε”* versus *f_0_*.

The test results for Q_u_ and f_o_ with different mole fractions of ethanol are shown in Figure 5. The solid and dashed lines represent measured and actual values, respectively. The values vary significantly when the ethanol concentration is over 80%; the value of Q_u_ decreases, whereas that of f_o_ increases with increasing ethanol concentration. The ethanol concentration can be estimated using (7), which can be employed for predictive analysis when sophisticated instruments are not available and the difference between the predicted and actual concentrations is no more than 10% [22]. The average percentage error between the theoretical and measured data can be calculated using (8).

(7) $$Ethanol\;concentration=(-3.4\times Q_{u}+304)\%$$

(8) $$\Delta =\frac{\sum_{i=1}^{m}\left|\frac{\varepsilon_{M}-\varepsilon_{A}}{\varepsilon_{A}}\right|}{m}\times 100\%$$

where ε_M_ and ε_A_ denote the measured and actual values of the relative complex permittivity, respectively, and m denotes the number of solution samples [23]. The percent error of the real part is 2.4% and that of the imaginary part is 2.5% when compared to the values in [24,25].

**Figure 6:**
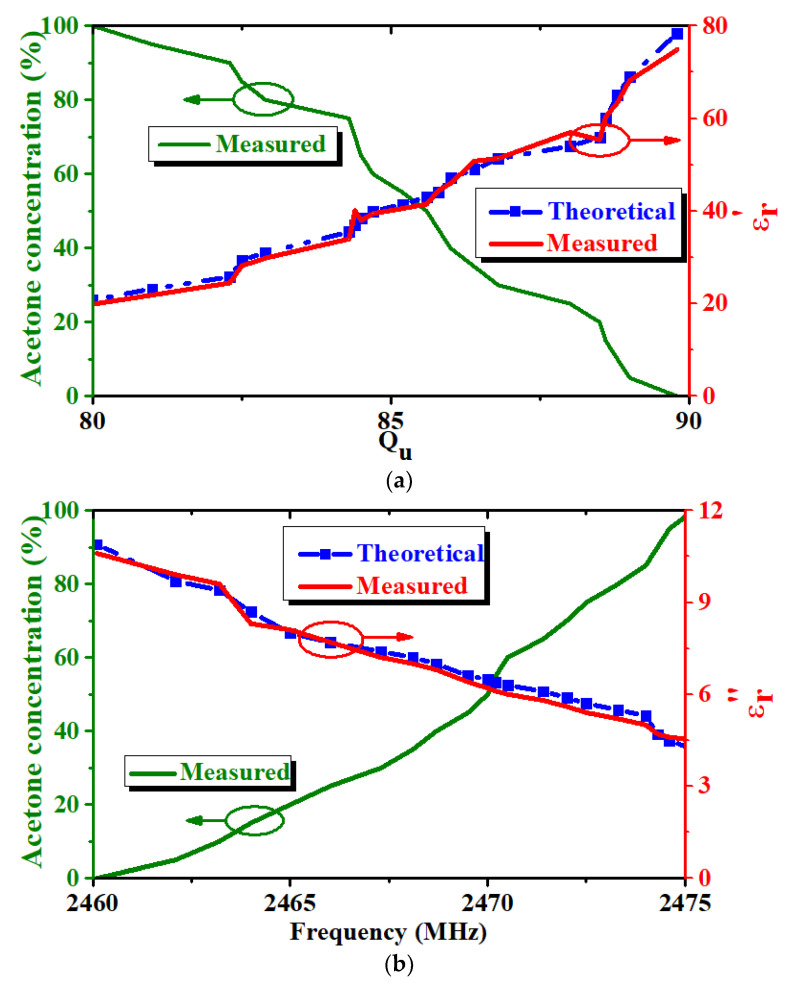
Test results of acetone–water mixtures: (**a**) Acetone concentration and *ε’* versus *Q_u_*; (**b**) Acetone concentration and *ε”* versus *f_0_*.

Figure 6 depicts the results of another test performed on an acetone–water mixture using the same method to verify the accuracy and sensitivity of the sensor. These values vary more minimally than those in the previous experiment because the permittivities of ethanol and acetone are different. The green line represents the variation in acetone concentration with respect to *Q_u_* or *f_0_*, and the red and blue colors represent the real and imaginary parts of the relative complex permittivity with respect to *Q_u_* and *f_0_*. The relative error of the real part is 2.5% and that of the imaginary part is 3.1% when compared with the values in [26]. *Q_u_* changes by approximately 0.1 for every 1% change in the concentration of the acetone. The desired results were achieved for ethanol and acetone detection with a relatively stable measurement error. The measured results indicate that the designed SIW sensor can respond quickly to the permittivity variation of the mixed solutions, indicating that the complex permittivity of a liquid with complex components can be measured. The designed sensor can be used to measure unknown liquids using the relationship between *Q_u_* and *f_o_* of the SIW resonator and the relative complex permittivity. 

## 4. Discussion

**Figure 7:**
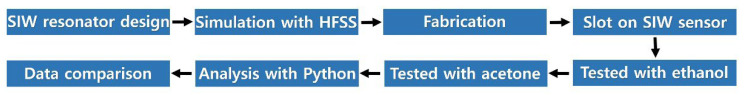
Sensor design and analysis flow.

The principles of measuring complex permittivity based on the SIW sensor are described in this article. As shown in Figure 7, the basic characteristics of the SIW were analyzed first in this study before introducing the working principle and Q-factor of the resonant cavity. The simulation results were used to optimize the dimensions. Finally, two different kinds of mixed solutions were tested, with the complex permittivities of the liquid mixtures derived using HFSS and Python. 

The SIW sensor was tested in air and liquid, with the corresponding calculations performed in a stepwise manner. As the relative permittivity of the gases is approximately 1, the influence of air is negligible. As a reusable and washable sensor, the designed SIW sensor can sensitively detect the output values and identify the liquid concentration. A relationship was derived to convert the measured data into the relative complex permittivity of the MUT. The sensor was validated based on an unloaded test to remove incorrect values, with the tests repeated thrice to determine average values [27,28]. Ethanol–water and acetone–water mixtures were selected to observe the changes in permittivity. Compared with other sensors used for sensing liquids in Table 2, the designed sensor in this work achieves good performance as well as a compact physical size of 79.2 mm × 59.8 mm, equivalent to the electrical size of 0.65λ_0_ × 0.49λ_0_, where λ_0_ denotes the wavelength in the free space at the operating frequency, with a planar SIW structure. The designed SIW resonator could work well in S-band with a sharp band edge. Measurement errors are primarily caused by the manufacturing process and test environment [29]. Additionally, a permittivity sensor can be used to measure the liquid permittivity in the L- or C-band by changing the size of the sensor [30,31]. 

## 5. Conclusions

With advances in efficiency and intelligent control, sensor applications have expanded beyond the traditional fields of pressure, temperature, and humidity. Scientists have developed a few real-time sensors to check the state of devices or areas of concern [36]. Considering the distribution of the electric field and temperature frequently change in the center slot of the substrate where the liquid is placed, an octagon-shaped sensor was designed in this study to measure the permittivity and observe its variation. The selected working frequency of the sensor is easy to control because it consists of only a one-port component [37,38]. The measured results show the relationships between the permittivity and parameters of the SIW sensor (tested at 25 °C). The influence of air humidity can be ignored because the sensor is partly immersed in the liquid, with the relative permittivity and permeability of the gas close to 1. SIW sensor performance was tested, and its applicability to liquids was verified. As non-invasive and contactless sensors, liquids can be tested efficiently and safely.

Technological progress has enabled more accurate sensors to be manufactured and applied more conveniently with more features. The use of sensing devices, including smart home sensors, increased rapidly with the advent of the global “trillion sensor” era [39]. Refrigerators, boilers, air conditioners, and laundry machines constitute a large percentage of the home appliance market share. The Coronavirus Disease 2019 (COVID-19) outbreak is currently restraining activities in the electrical industry due to supply chain disruption, and a decline in purchasing power and the adoption of smart home appliances and electric vehicles among consumers will further hinder global market growth. However, the post-COVID-19 impact will accelerate market growth and promote the development of intelligent technologies in the near future [40]. Permittivity sensors can be installed in the drains of home appliances to detect liquid quality, and a smartphone application can be built for real-time data collection. In the future, a more compact structure with high efficiency for accessing unreachable materials, including solids, will be studied, and a higher-performance algorithm will be developed [41]. Further research on developing devices with better hydrophobic performance, enhanced precision, and advanced processing technology will also be investigated.

## Figures and Tables

**Table 1:** Test results of SIW resonator with water at different temperatures.

Temperature (℃)	*f_0_* (MHz)	*Q_u_*
30	2453.7	87.5
25	2460.0	89.2
20	2467.2	92.1

**Table 2:** Comparison with other liquid sensors.

Reference	Structure	Frequency (GHz)	Relative Size (λ_0_ × λ_0_)	Error
[32]	Split-ring resonator	2.5	0.15 × 0.15	6.5%
[33]	Open-loop resonator	2.6	>0.18 × 0.09	6.3%
[12]	Square SIW	2.19	0.4 × 0.36	3.2%
[29]	Circular SIW	4.4	1.17 × 1.17	0.9%
[30]	Stepped impedance resonator	1.91	0.26 × 0.2 [33]	4.5%
[4]	SIW using 3-D printing	3.82	0.66 × 0.66	11.6%
[34]	Multiple complementary split-ring	2.45	0.27 × 0.2	4.9%
[31]	Quarter mode SIW	4.55	0.91 × 091	3.9%
[35]	SIW re-entrant cavity resonator	2.29	0.49 × 0.49	7.8%
[13]	Dual-band SIW	2.45/5.85	>1.05 × 0.72	2.7%
This work	Octagon-shaped SIW	2.45	0.65 × 0.49	3.1%

## Data Availability

Not applicable.
